# Procedural Pain and Situational Anxiety in Pediatric Patients: A Narrative Review of Assessment Tools

**DOI:** 10.3390/pediatric18010005

**Published:** 2026-01-02

**Authors:** Anna Wojciechowska-Urbanek, Jowita Rosada-Kurasińska, Alicja Bartkowska-Śniatkowska

**Affiliations:** 1 Institute of Health and Physical Culture, Jan Amos Comenius Academy of Applied Sciences in Leszno, 5 Adam Mickiewicz Street, 64-100 Leszno, Poland; aniawojciechowska1@wp.pl; 2 Department of Paediatric Anesthesiology and Intensive Therapy, Poznan University of Medical Sciences, Fredry 10, 61-701 Poznan, Poland; asniatko@ump.edu.pl

**Keywords:** pediatric patients, procedural pain, situational anxiety, pain assessment

## Abstract

**Background**: Procedural pain and anxiety are common phenomena among children hospitalized in pediatric wards; however, they are often under-recognized. These experiences frequently accompany patients during various diagnostic and therapeutic procedures. Assessing pain is particularly challenging in pediatric care, as children may have difficulty clearly expressing their pain experiences, which can result in the underestimation of their symptoms. Accurate assessment using an appropriately selected scale should be regarded as an essential component of high-quality healthcare. **Methods**: This narrative review summarizes standardized tools for assessing procedural pain and anxiety in pediatric patients, focusing on instruments adapted to different ages and cognitive abilities and on their applicability in everyday clinical practice. **Results:** Numerous standardized scales are available, ranging from behavioral to numerical instruments tailored to specific developmental stages. Despite this, the implementation of these tools in routine care remains inconsistent, largely due to organizational, educational, and communication barriers among healthcare professionals. **Conclusions**: Medical staff must not only receive adequate training but also exhibit the motivation and readiness to utilize available pain assessment methods. Education and increased awareness among staff regarding pain and procedural anxiety are crucial for improving patient comfort and safety.

## 1. Introduction

The hospitalization of children in pediatric wards is associated with repeated exposure to painful and stressful stimuli. Pain most often arises from medical procedures, such as surgery, or originates from the underlying disease itself. Although the earliest known documents detailing attempts to administer analgesia date back more than 3500 years to ancient Egypt, widespread belief persisted until the latter half of the twentieth century that young children did not experience pain, allegedly due to the immaturity of their central nervous system. As a result, pain in children was frequently undertreated, which negatively impacted their emotional and psychological development and could manifest as withdrawal, irritability, anxiety, and distress. It was not until 1995, following the initiative of the American Pain Society, that pain was formally recognized as the fifth vital sign. Although pain perception is shaped by physical factors, emotional influences, and the child’s personality, accurate assessment of pain intensity enables the selection of appropriate therapeutic interventions and the evaluation of their effectiveness. Currently, it is estimated that more than 40 scales exist for assessing acute pain intensity in young children, alongside multidimensional scales and questionnaires for evaluating pain in older children. Consequently, pain management should be viewed as a priority and the gold standard in pediatric care.

Procedural pain and anxiety can amplify each other through shared neurobiological and behavioral pathways; anxiety may increase pain perception or mask it in some children. In routine practice, pain and anxiety should usually be assessed with distinct but complementary tools, while in specific contexts (pre-procedural settings, children with communication difficulties), integrated or hybrid approaches that simultaneously capture pain-related distress should be adopted.

The aim of this review is to provide an overview of selected methods for the assessment of procedural pain and situational anxiety in a pediatric patient population, regardless of their age or intellectual level.

## 2. Methods

We provide an overview of up-to-date evidence drawn from clinical research, review articles, and professional guidelines. This article was conceived as a narrative, educational review rather than a systematic review or meta-analysis. The literature search was conducted in PubMed, complemented by a hand search of reference lists of key articles and relevant clinical guidelines. The following keywords and their combinations were used: “pediatric pain”, “procedural pain”, “anxiety”, “pain assessment”, “anxiety scales”. The search covered articles published between 2000 and 2025 in English and Polish. To minimize the risk of omitting important tools, we cross-checked our selection against recent international guidelines and major review articles on pediatric pain and anxiety assessment, and prioritized instruments with psychometric validation and documented clinical use [1,2].

## 3. Selected Pain Assessment Instruments Utilized in Neonatal Populations

An unquestionable challenge for medical personnel is the assessment of pain perception in the youngest group of patients—premature infants and newborns. These difficulties arise primarily from the lack of verbal communication with the patient, which necessitates continuous observation of the infant’s body and behavioral changes such as muscle tension, facial grimacing, altered body posture, or clenched fists. Pain in newborns may occur not only during invasive procedures involving skin disruption but also during routine care activities [3]. It should be emphasized that the nerve endings and mechanisms of pain perception in newborns are comparable to those in adults. Moreover, premature infants possess an immature system for inhibiting pain perception, which leads to increased sensitivity to painful stimuli. Exposure of premature babies to repeated pain may result in gradual sensitization and the development of adverse pain-related responses. It should also be noted that painful procedures in newborns and premature infants are often repeated rather than limited to a single instance. Prolonged exposure to painful stimuli and the resulting stress may negatively affect the future development of a newborn, both during childhood and later in adulthood, as it can lead to emotional and behavioral disorders as well as impaired cognitive functions [4]. To date, numerous tools and scales have been developed to assess pain in this age group; however, due to the complex pathomechanism of pain, it has not been possible to identify a single indicator that clearly reflects the level of pain intensity. Scales based on the measurement and monitoring of physiological parameters—such as heart rate, blood pressure, oxygen saturation (SpO_2_), and transcutaneous partial pressure of oxygen or carbon dioxide—are commonly used. Changes in behavior induced by pain are evaluated using various behavioral assessment tools [3].

A widely used behavioral scale for assessing the intensity of procedural pain in newborns and premature infants is the Neonatal Facial Coding System (NFCS). This scale evaluates characteristic changes in an infant’s facial expression that are indicative of a pain response. It focuses on seven indicators, each observed and coded on a binary (0–1) scale. These indicators represent classic signs of pain: frowning, brow bulging, nose wrinkling, mouth opening, horizontal mouth stretching, and perioral tension. The higher the total score assigned to an infant, the greater the likelihood of a strong pain response [5].

Another tool designed to assess pain in newborns is the Premature Infant Pain Profile (PIPP) scale, which evaluates seven parameters: gestational age, behavioral state, brow bulge, eye squeeze, nasolabial furrow deepening, heart rate, and oxygen saturation. The infant’s condition is recorded for 45 s, with physiological parameters monitored every 3 s. The video recording is subsequently analyzed to determine pain intensity. A lower total score indicates a lower level or absence of pain [6].

One of the scales that focuses on both physiological and behavioral changes is the Pain Assessment Tool (PAT), developed by Hodgkinson et al. in 1994 (Table 1) [7]. It is used to assess the intensity of pain, including procedural pain following surgical interventions, and can be applied to newborns born after 33 weeks of gestation. The tool includes nine parameters: body posture, sleep state, facial expressions, crying, skin color, respiratory pattern, oxygen saturation, heart rate, and blood pressure. Observation of the infant’s behavior should last approximately 20 s, after which vital signs are measured. Pain assessment in postoperative newborns is conducted immediately after surgery and subsequently every hour during the day. On the first postoperative day, pain perception is evaluated every 2 to 4 h [7]. The underlying principle of this scale is to rely on the nurse’s perception and interpretation of the newborn’s pain-related signs. Each parameter in the PAT scale is scored from 0 to 2, where 0 indicates no pain and 2 represents the maximum level of pain. Until an appropriate level of analgesia is achieved, pain intensity should be monitored every 30 min and then hourly thereafter. A score ranging from 0 to 5 suggests no or minimal pain, while scores between 6 and 10 indicate mild to moderate pain. Scores in the range of 10 to 20 reflect severe pain sensations [7,8].

In neonatal intensive care units, the standard tools for assessing pain and the level of sedation in pediatric patients are the COMFORT scale and its modified version, COMFORT-B. The scale was developed by Ambuel et al. in 1992 to provide an objective assessment of discomfort, pain, and sedation in children requiring respiratory support or anesthesia [9]. Each parameter is rated on a five-point scale, and the total score indicates the intensity of pain and the degree of sedation. The COMFORT-B scale is a behavioral clinical tool comprising six components: alertness, calmness or agitation, respiratory response (or crying in patients not receiving mechanical ventilation), physical movement, muscle tone, and facial tension. Each item is rated on a 5-point scale, resulting in a total score ranging from 6 to 30. Scores between 6 and 10 suggest oversedation, scores from 11 to 23 indicate moderate sedation, and scores from 24 to 30 reflect minimal sedation (Table 2) [10]. This adaptation focuses solely on behavioral indicators, enhancing the scale’s practicality and reliability in routine clinical practice. It is recommended by international anesthesiology and neonatology societies as a primary tool for assessing pain in children in intensive care settings [9,11,12].

Among the recommended tools for assessing pain intensity in newborns is the CRIES scale (Crying, Requires O_2_, Increased vital signs, Expression, Sleepiness), developed by Krechel et al. in 1995 [13]. This scale allows for an objective evaluation of postoperative pain in newborns based on five behavioral and physiological indicators: crying, the need for oxygen supplementation, changes in vital signs compared with preoperative values, facial expression, and sleep or wakefulness state. The total score ranges from 0 to 10 and helps determine the need for pain management interventions; a score above 4 indicates the necessity of pharmacological analgesia [12].

The N-PASS (Neonatal Pain, Agitation, and Sedation Scale), developed by Hummel and Puchalski in 2004 [7], is used to assess pain and the level of sedation in mechanically ventilated newborns [9]. The purpose of this tool is to provide an objective, consistent, and practical evaluation of an infant’s response to painful stimuli, thereby supporting clinical decision-making regarding analgesia and sedation in neonatal intensive care units. The N-PASS assesses five behavioral and physiological parameters: crying or irritability, behavior state, facial expression, muscle tone, and physiological indicators such as heart rate, blood pressure, respiration, and oxygen saturation. Each parameter is scored from −2 to +2 points. A total score between −10 and −5 indicates deep sedation, while a score from −5 to −2 reflects light sedation. It is recommended that sedation levels in children be assessed every 2–4 h and additionally after the administration of an analgesic. When assessing pain, positive values of the scale are applied. If the total score is 3 or higher, analgesic treatment should be initiated. Postoperative pain monitoring should be carried out every 2 h during the first two days. In this scale, the level of sedation does not need to be assessed simultaneously with the level of analgesia. Numerous studies have confirmed the usefulness and reliability of the N-PASS as a tool for evaluating pain in invasively ventilated infants [9].

The use of the aforementioned scales requires careful patient observation, clinical experience of medical staff, and is not a continuous process. Currently, the Newborn Infant Parasympathetic Evaluation (NIPE) monitor is gaining attention as an innovative tool for pain assessment. The operation of the NIPE monitor is based on the analysis of data obtained from standard patient monitoring systems. It was developed to overcome the limitations of traditional pain assessment scales and has been validated for use in children under two years of age. The device measures high-frequency heart rate variability (HRV) (0.15–0.40 Hz) as an indicator of parasympathetic nervous system (PNS) activity and provides an objective pain assessment on a scale from 0 to 100, where lower values correspond to higher pain intensity. As a modern technology, the NIPE monitor can be employed to evaluate prolonged pain during procedures performed without anesthesia. The use of the NIPE monitor also enables the assessment of acute pain and the optimization of environmental conditions surrounding the patient, such as noise, lighting, and body positioning. Owing to its capacity for continuous monitoring, this device may serve as a valuable adjunct in the evaluation of procedural pain, analgosedation, and postoperative analgesia. It offers an objective method for assessing the comfort of newborns treated in intensive care units. It should be emphasized that any advancement improving pain monitoring in newborns is of great clinical importance [3].

## 4. Selected Pain Assessment Instruments Utilized in Children with Disabilities

Another challenge faced by the therapeutic team is the assessment of pain in patients with intellectual disabilities. Behavioral abnormalities may interfere with the evaluation of parameters such as facial expressions, body movements, motor stereotypes, seizure activity, or idiosyncratic behaviors. Although self-assessment is considered the gold standard in pain evaluation, its reliability is limited in individuals with intellectual disabilities, which justifies the use of alternative assessment methods. The most commonly used scales for this group of patients are the PPP (Pediatric Pain Profile), NCCPC-R (Non-Communicating Children’s Pain Checklist-Revised), FLACC (Face, Legs, Activity, Cry, Consolability), and the R-FLACC, developed by Malviya et al. [14].

A simple and user-friendly observational tool is the FLACC scale, originally developed for young children but also applicable to patients with intellectual disabilities. Pain assessment using this scale takes only a few minutes and can be effectively used to evaluate procedural, postoperative, and acute pain intensity. The scale includes five parameters: leg position, facial expression, activity, crying, and consolability. Each parameter is scored on a scale of 0, 1, or 2 points. The total score, ranging from 0 to 10, reflects the overall pain level, where 0 indicates no pain and 10 represents severe pain. According to the literature, scores between 4 and 6 points suggest the need for analgesic intervention, while results above 7 points warrant immediate pharmacological pain management (Table 3) [15]. The R-FLACC scale additionally takes into account the patient’s individual behaviors [14].

When assessing pain in children with disabilities, an essential aspect of the therapeutic team’s work is effective collaboration with the child’s primary caregivers, who have valuable experience and are best able to interpret their child’s behaviors and needs. One of the tools developed for assessing pain in disabled or non-verbal children aged 3 to 18 years is the NCCPC-R (Non-Communicating Children’s Pain Checklist–Revised) scale relies on reports from caregivers familiar with the child’s behavioral responses to pain [16]. Based on this information, a list of the 30 most common pain-related behaviors was developed, grouped into seven categories: vocalization (sounds made), social behavior (irritability or calmness), facial expressions, physical activity (immobility or increased movement), muscle tone of the body and limbs, physiological indicators (breathing pattern, skin color), and eating and sleeping patterns. Pain assessment is conducted every two hours, and each observed behavior is scored from 0 (no change) to 3 (frequent occurrence). A total score of 7 points or more is considered indicative of the presence of pain.

A similar tool is the Pediatric Pain Profile (PPP) scale, which is based on a set of 20 pain-related behaviors encompassing facial expressions, body movements, muscle tone, social reactions, and mood. These behaviors are observed over a five-minute period and rated on a 0–3 point scale. A total score above 14 points indicates the presence of pain [15].

Another tool for monitoring pain in individuals with intellectual disabilities, particularly those with multiple disabilities, is the Douleur Enfant San Salvadour (DESS) scale. To assess the intensity of pain, it is first necessary to gather information about ten behaviors exhibited under the patient’s normal functioning conditions. According to the data collection questionnaire, these behaviors focus on the child’s screaming or crying, motor reactions, responses to touch, facial expressions, level of interest in the surroundings, interaction with adults, and adoption of a comfortable body position. After obtaining baseline information about the child’s usual behavior, the evaluator assesses pain at eight-hour intervals using the scale, assigning scores of 0, 1, 2, 3, or 4 to specific categories. The total score ranges from 0 to 40 points, with pharmacological treatment recommended when the patient reaches the score of 6 points [16].

A promising new technology for objectifying pain assessment in individuals with intellectual disabilities is the measurement of skin conductance fluctuations using a Skin Conductance Algesimeter (SCA). Skin conductance serves as an indicator of sympathetic nervous system activation in response to a pain stimulus. This method can be applied to objectively evaluate pain perception in children, including preterm infants, as well as in postoperative or mechanically ventilated patients [17].

## 5. Selected Pain Assessment Instruments Utilized in Older Children

Pain assessment is a fundamental component of appropriate clinical management in pediatric care. Consequently, reliable assessment methods have been developed to accurately reflect a child’s response to painful stimuli. One simple and user-friendly tool for evaluating pain in children aged 3 to 12 years is the Finger Pain Scale. This method can also be applied to patients with language barriers. The concept of the scale is based on the distance between the index finger and the thumb—the greater the span, the higher the intensity of the perceived pain stimulus.

A widely used scale in clinical practice is the Wong–Baker FACES Pain Rating Scale, designed for children aged 3 to 7 years [18]. Patients in this age group can identify the intensity of their pain using illustrated facial expressions, enabling an assessment that combines self-observation with self-reporting. The Wong–Baker scale allows for both visual and emotional evaluation of pain. It consists of six facial drawings ranging from a smiling face, representing no pain, to a crying face, indicating the most severe pain, allowing the child to select the face that best represents their current state [19].

For children over 8 years of age, numerical scales are commonly used to assess pain intensity. These tools require the child to understand and interpret numeric rating systems. The most frequently applied methods are the Visual Analog Scale (VAS) and the Numerical Rating Scale (NRS). Both scales involve assigning a value from 0 to 10 on a horizontal line, where 0 represents no pain and 10 indicates the highest possible level of pain [20].

A summary of the above-mentioned pain assessment tools used in pediatric patients is presented in Table 4.

## 6. Fear of Pain in Pediatric Patients

Fear of pain is a significant problem among hospitalized children, adversely affecting their well-being and the treatment process. Fear of medical procedures and the associated pain often cause severe stress, which may lead to anxiety disorders related to separation from parents, bedwetting, eating disturbances, insomnia, aggression, or avoidance of healthcare. This fear not only impacts the child but also affects their parents and the entire care team. Parental anxiety regarding procedures is a crucial factor influencing how a child responds to pain, with parental distress potentially intensifying the child’s anxiety. However, appropriate emotional support and positive parental attitudes can foster effective coping mechanisms for pain and stress. Addressing fear of pain through psychological support, family involvement, and targeted pain management strategies is essential for improving outcomes in hospitalized children [21]. Many studies have demonstrated familial aggregation of anxiety, supporting the view that if parents are diagnosed with anxiety related to hospitalization, the risk of anxiety in their children increases significantly. Research by Lebowitz et al. highlights the intergenerational transmission of fear, indicating that children may learn to be afraid by observing their parents’ emotional reactions. Additionally, a meta-analysis by Hettema et al. [22] estimates that genetic heritability accounts for 30–40% of various anxiety disorders, representing a major source of familial anxiety occurrence. These findings underscore both environmental and genetic contributions to anxiety transmission within families, emphasizing the importance of addressing parental anxiety to mitigate its impact on children [23].

Anxiety, regardless of its origin, negatively impacts the health and quality of life of hospitalized children, leading to significant consequences for both the patients and their families. Symptoms such as generalized anxiety and fear related to hospital environments can persist for up to 12 months following medical procedures. Needle phobia is particularly common, affecting up to two-thirds of children and often resulting in avoidance of essential vaccinations. Fear experienced during pediatric medical care often leads to avoidance of healthcare in adulthood and neglect of preventive health measures.

To prevent these adverse outcomes, targeted interventions and standardized, validated assessment tools must be implemented to effectively identify and treat anxiety in children. Addressing anxiety through psychological support, family involvement, and tailored pain management is crucial to improving long-term health outcomes and quality of life for hospitalized children [24].

## 7. Selected Tools for Measuring Situational Anxiety

Although fear and anxiety are natural adaptive responses, in medical settings they may intensify into excessive anxiety, impairing patient compliance and hindering the therapeutic process. Consequently, the use of validated anxiety assessment scales is essential for minimizing the adverse effects of anxiety and optimizing treatment outcomes.

The Visual Analog Scale (VAS) and the Facial Anxiety Scale (FAS) are widely used tools for assessing anxiety associated with hospitalization, as well as needle-related, diagnostic, and therapeutic procedures. Both scales rely on patient self-report and are applied to evaluate procedural anxiety, mood disturbances, quality of life, and pain.

The Visual Analog Scale (VAS) consists of a 10 cm line labeled with descriptions of emotional states ranging from ‘I don’t feel anxious at all’ to ‘the most anxious I can imagine.’ The scale may also include color coding to facilitate the interpretation of the patient’s emotional experience. During the assessment, the patient is asked to mark the point that best reflects their current level of anxiety. Scores between 0 and 3 indicate mild anxiety, while scores from 7 to 10 suggest severe anxiety. Despite its simplicity, the VAS has certain limitations. It is suitable for use in children aged 7 years and older, as younger children may have difficulty understanding the meaning of the verbal anchors. This can lead to misinterpretation and inaccurate self-assessment of their emotional state [24].

Another easy-to-use tool is the Facial Anxiety Scale (FAS), which consists of nine faces representing different emotional states. The patient is asked to select the face that best reflects their current feelings. This scale is widely used because it helps children better conceptualize and express their emotions. Like the VAS, it is designed for older children who are capable of identifying and describing their emotional states [24].

To facilitate the rapid assessment of anxiety during anesthesia induction, clinicians have developed the Pediatric Anesthesia Behavior Scale (PAB). This scale categorizes children’s behaviors as happy, sad, or frantic, assigning corresponding scores from 1 to 3. However, its simplicity comes with limitations, notably the lack of evaluation of patient–staff cooperation. A child’s display of sadness or anxiety does not necessarily imply noncooperation. In the perioperative context, accurately distinguishing between emotional expression and cooperative behavior is essential, as overinterpreting facial cues may lead to inappropriate clinical judgments [24].

A comparable observational tool for evaluating anxiety and behavior in children during anesthesia induction is the Induction Compliance Checklist (ICC). This scale comprises a checklist of ten items that capture negative behaviors observed during induction, such as crying, physical resistance, or attempts to escape. The ICC provides a standardized method for assessing the patient’s level of compliance as well as the presence of resistant and anxious behaviors. Scoring is binary, with each observed behavior assigned 1 point and its absence 0 points; the total score is the sum of all items (maximum score: 10). A score of 0 indicates an optimal induction of anesthesia, scores from 1 to 4 reflect suboptimal induction, and scores above 4 denote poor induction [25].

Another observational tool for assessing anxiety is the HARD± scale. This instrument evaluates the child’s emotional state across domains such as happiness, relaxation, anxiety, and worry using yes/no responses, while also assessing cooperation and adherence to instructions [25].

The most widely used instrument for assessing procedural or preoperative anxiety is the Modified Yale Preoperative Anxiety Scale (m-YPAS). This scale comprises 22 items divided into five behavioral domains and relies on observer-based assessment at multiple time points. It evaluates parameters such as activity, vocalization, emotional expressivity, state of arousal, and interactions with family members. Each parameter is rated on a 1–4 or 1–5 scale, depending on the item. Owing to its complexity, a shortened version—the m-YPAS Short Form (m-YPAS-SF)—was developed, encompassing four domains assessed at two time points. In both versions, an anxiety level is defined as a score of 30 or higher. The overall score is calculated using an algorithm that integrates the values assigned to individual behavioral categories [24]. The m-YPAS is regarded as the gold standard for evaluating preoperative anxiety in children

The Observation Scale of Behavioral Distress (OSBD) was developed to assess children’s behavioral responses during medical procedures. The scale includes 11 behavioral items, which are summed and then divided by the duration of the procedure to yield a distress score. It has been used primarily to evaluate anxiety, particularly procedural anxiety. However, its application in real-world clinical settings is challenging because it requires video recording of procedures to reliably code the child’s behavior and accurately determine procedure duration. Owing to this complexity, the OSBD has not been widely adopted in routine clinical practice [24].

Another commonly used instrument for measuring perioperative anxiety in children is the State-Trait Anxiety Inventory for Children (STAIC). It is designed for literate children aged approximately 5–16 years, who must be able to read and understand the questionnaire items. Completing the inventory typically takes about 5–10 min.

The STAIC comprises 20 items assessing state anxiety (situational, transient anxiety) and 20 items assessing trait anxiety, reflecting a general tendency to experience fear and anxiety. Scores of around 20 points indicate low anxiety, whereas scores approaching 60 points indicate high anxiety. Final scores are interpreted using percentile ranks and Z scores adjusted for the child’s age and gender. To gain additional perspective on the severity of anxiety symptoms, the STAI (State-Trait Anxiety Inventory) scale, completed by one of the parents, can be used. The scale assesses parental anxiety. Twenty items address parental anxiety in state blocks, and 20 items assess trait anxiety. Scores range from 0 to 60, and interpretation is based on the appropriate percentiles [26,27].

The main characteristics of each method are listed in Table 5.

## 8. Barriers to Implementation

Pain and anxiety assessment in children is a crucial component of medical care, yet its effective implementation in clinical practice still faces numerous challenges. Despite the availability of standardized assessment scales, their use in pediatric patients remains limited by organizational, educational, and communication barriers.

Organizational barriers constitute major obstacles to the systematic assessment of pain in children. The most reported factors include staffing shortages, excessive workloads among healthcare professionals, and the resulting time constraints. In addition, the absence of consistent institutional policies and appropriate organizational structures limits the effective implementation and maintenance of pain assessment standards. Poor communication within treatment teams and the marginalization of pain as a therapeutic priority further exacerbate these difficulties. The lack of structured protocols, limited interprofessional collaboration, and reluctance to use non-pharmacological methods of pain management also remain important problems, ultimately hindering the delivery of consistent, comprehensive, and effective pediatric care.

Educational barriers are another important factor limiting the effective implementation of pain assessment and treatment in children. Unfortunately, nursing staff often have insufficient knowledge of pain mechanisms and the principles of pain management in the pediatric population. And it should also be noted that acute pain is among the most frequently identified nursing diagnoses in hospitalized children [28]. Limited familiarity with available tools, combined with the absence of a single universal instrument for assessing pain in children, hinders their appropriate selection and consistent use in clinical practice. In addition, misconceptions about children’s ability to experience and communicate pain, as well as limited confidence in the reliability of pain assessment scales, further reduce their use. Therefore, the need for systematic and continuous education of medical staff as a key element in improving the quality of pain assessment and treatment in children is emphasized.

Another aspect that hinders pain assessment in patients is communication barriers with both the child and the parent. Unlike adults, pediatric patients often express pain indirectly, and their ability to convey information accurately is limited by their stage of cognitive and emotional development. These difficulties can lead to underestimation of pain intensity and misinterpretation of its symptoms by medical staff. An additional challenge is the role of parents or guardians, who sometimes underestimate a child’s pain or misinterpret their behavior. In the stressful and confusing circumstances of hospitalization, they may provide inconsistent or inaccurate information to the medical team. In addition, some parents express concerns about the potential side effects of painkillers or their routes of administration, which may limit the effectiveness of treatment. Children’s resistance to taking medication, especially unpleasant-tasting preparations, is an additional barrier to effective pain management.

In addition, technological barriers may exist in hospital settings that limit the effectiveness of pain assessment and monitoring in children. These include outdated technological infrastructure and inequalities in access to modern tools to support pain assessment, which hinders the standardization of clinical practice.

Psychosocial factors are also an important aspect, especially in adolescent patients. Teenagers may fear stigmatization, the need to distinguish themselves from their peers or being perceived as weaker, which leads to underreporting of the level of pain they experience. These phenomena further complicate the process of reliable pain assessment and may result in inadequate treatment [29].

## 9. Limitations

The current body of evidence is largely based on small, single-center studies that use diverse and often non-standardized methodologies, underscoring the need for adequately powered multicenter trials. Larger studies using harmonized protocols and clearly defined decision thresholds would enhance reproducibility, and there is a particular need to test multimodal, clinically integrated assessment pathways. Pediatric and neonatal populations remain especially under-represented and require robust validation studies, including cost-effectiveness evaluations, before broader implementation can be recommended.

## 10. Conclusions

It is now widely acknowledged that pain in children remains an underestimated phenomenon, even though pain relief should be a priority in the diagnosis and treatment of all patients. It ought to be a standard of care rather than an exceptional measure. Inadequate management of procedural pain can lead to trauma, fear of future medical procedures, and diminished trust in medical personnel. Despite the availability of various tools for assessing pain intensity and opportunities to mitigate it, children continue to experience its adverse effects. Procedural pain remains a frequent occurrence in hospital settings, and children are often exposed to painful interventions repeatedly. Yet effective implementation of pain assessment in clinical practice still faces numerous challenges. Despite the availability of standardized pain assessment scales, their use in pediatric patients remains limited by organizational, educational, and communication barriers.

To improve the quality of pain assessment and treatment in children and to overcome organizational, educational, communication, and technological barriers, it is necessary to implement multifaceted interventions at the level of pediatric wards. It is crucial to ensure adequate staffing and rational work organization to allow for systematic, regular, and well-documented pain assessment in all pediatric patients. An important element in improving the quality of care is the provision of ongoing training for healthcare professionals, particularly nurses, on pain mechanisms, pain management strategies, and the appropriate selection of tools for assessing pain in children. Strengthening communication within therapeutic teams and fostering interprofessional collaboration through regular meetings, joint treatment planning, and consistent medical documentation also appears essential. Actively involving parents or caregivers in the process of pain assessment and treatment, together with clear education on the safety of the methods used and the importance of reporting pain symptoms, can further enhance the effectiveness of therapy.

Unfortunately, planned procedures are rarely preceded by deliberate and targeted pain relief measures. Addressing this issue requires the dedication, commitment, time, and empathy of medical staff. Therefore, appropriate training of healthcare professionals on pain assessment is fully justified, as it enhances sensitivity to the needs of the youngest patients and significantly improves the quality of care.

## Figures and Tables

**Table 1:** Neonatal Pain Assessment Tool.

Parameter	Reaction	Points
Posture/Tone	Flexed and/or tense	2
	Extended	1
Sleep Pattern	Agitated or with drawn	2
	Relaxed	1
Expression	Grimace	2
	Frown	1
Cry	Yes	2
	No	0
Color	Pale/Dusky/Flushed	2
	Pink	0
Respirations	Apnea	2
	Tachypnoea	1
Heart Rate	Fluctuating	2
	Tachycardia	1
Saturations	Desaturating	2
	Normal	0
Blood Pressure	Hypotensive/Hypertensive	2
	Normal	0
Nurses Perception	Yes Pain	2
	No Pain	0
Total Score		

**Table 2:** COMFORT-B Scale.

Parameter	Reaction	Points
Alertness	Deeply asleep	1
	Lightly asleep	2
	Drowsy	3
	Fully awake and alert	4
	Hyperalert	5
Calmness/agitation	Calm	1
	Slightly anxious	2
	Anxious	3
	Very anxious	4
	Panicky	5
Respiratory response (ventilated children)	No coughing and no spontaneous respiration	1
	Spontaneous respiration with little or no response to ventilation	2
	Occasional cough or resistance to ventilator	3
	Actively breathes against ventilator or coughs regularly	4
	Fights ventilator, cough or choking	5
Cry (non-ventilated children)	Quiet breathing, no crying	1
	Sobbing or gasping	2
	Moaning	3
	Crying	4
	Screaming	5
Physical movement	No movement	1
	Occasional, slight movements	2
	Frequent, slight movements	3
	Vigorous movement limited to extremities	4
	Vigorous movements including torso and head	5
Muscle tone	Muscles totally relaxed, no muscle tone	1
	Reduced muscle tone	2
	Normal muscle tone	3
	Increased muscle tone and flexion of fingers and toes	4
	Extreme muscle rigidity and flexion of fingers and toes	5
Facial tension	Facial muscle totally relaxed	1
	Facial muscle tone normal; no facial muscle tension evident	2
	Tension evident in some facial muscles	3
	Tension evident throughout facial muscles	4
	Facial muscles contorted and grimacing	5

**Table 3:** FLACC scale.

Criteria	Score 0	Score 1	Score 2
Face	No expression or smile	Occasional grimace/frown, withdrawn or disinterested	Frequent to constant quivering chin, clenched jaw
Legs	Normal position or relaxed	Uneasy, restless, tense	Kicking or legs drawn up
Activity	Lying quietly, normal position, moves easily	Squirming, shifting back and forth, tense	Arched, rigid or jerking
Cry ventilated children non-ventilated children	No cry No cry (awake or asleep)	Facial expressions: moaning or whimpering, occasionally complaining Moans or whimpers, occasional complaint	Facial expressions; crying steadily, screaming or sobbing, frequent complaints Cry steadily, screams or sobs, frequent complaint
Consolability	Content, relaxed	Reassured by occasional touching, hugging or being talked to, distractable	Difficult to console or comfort

**Table 4:** Comparison of the pain assessment tools.

Scale	Target Population	Practical Applications	Components	Scoring Range
NFCS (Neonatal Facial Coding System)	Newborn	Procedural pain	Behavioral (frowning, brow bulging, nose wrinkling, mouth opening, horizontal mouth stretching, and perioral tension)	0–7
PIPP (Premature Infant Pain Profile)	Preterm and term neonates	Procedural pain	Facial actions (brow bulge, eye squeeze, nasolabial furrow), Heart rate change, Oxygen saturation change, Gestational age/behavioral state	0–21
PAT (Pain Assessment Tool)	Infants and newborn	Procedural pain and Intensive Care Unit, newborn Department	Behavioral (body posture, sleep state, facial expressions, crying, skin color, respiratory pattern, oxygen saturation, heart rate, and blood pressure)	0–20
COMFORT/COMFORT-B	Hospitalized infants/children	Procedural pain and ongoing pain	Behavioral (facial tension, movement, crying/alertness) and physiological items (breathing, Heart rate)	6–30
CRIES (Crying, Requires O_2_, Increased vital signs, Expression, Sleeplessness)	Newborn	Procedural pain	Behavioral and physiological indicators: crying, need for oxygen supplementation, changes in vital signs compared with preoperative values, facial expression, and sleep or wakefulness state.	0–10
N-PASS (Neonatal Pain, Agitation, and Sedation Scale)	Infants and newborn	Procedural pain, ongoing pain and sedation for Intensive Care Unit	Behavioral and physiological parameters: crying or irritability, behavior state, facial expression, muscle tone, and physiological indicators such as heart rate, blood pressure, respiration, and oxygen saturation.	±10
PPP (Pediatric Pain Profile)	Children with severe disabilities	Chronic pain	20 pain-related behaviors encompassing facial expressions, body movements, muscle tone, social reactions, and mood	0–60
NCCPC-R (Non-Communicating Children’s Pain Checklist, Revised)	Disabled or non-verbal children	Acute and chronic pain	A list of the 30 most common pain-related behaviors: vocalization (sounds made), social behavior (irritability or calmness), facial expressions, physical activity (immobility or increased movement), muscle tone of the body and limbs, physiological indicators (breathing pattern, skin color), and eating and sleeping patterns.	0–90
FLACC (Face, Legs, Activity, Cry, Consolability)	Infants and young children unable to self-report	Procedural pain and use for procedures	Face, Legs, Activity, Cry, Consolability	0–10
DESS (Douleur Enfant San Salvadour)	Children with disabilities	Acute and chronic pain	Information about ten behaviors exhibited under the patient’s normal functioning conditions or crying, motor reactions, responses to touch, facial expressions, level of interest in the surroundings, interaction with adults, and adoption of a comfortable body position	0–40
Finger Pain Scale	Small children and patients with language barriers	pain	Distance between the index finger and the thumb—the greater the span	---
Wong–Baker Faces Pain Raining Scale	Older children	Self-report during procedures	Child selects a face/number representing pain intensity	0–10
VAS (Visual Analog Scale) and NRS (Numeric Rating Scale)	Older children	Self-report during procedures	Assigning a value from 0 to 10 on a horizontal line	0–10

**Table 5:** Comparison of the tools for measuring situational anxiety.

Scale	Target Population	Practical Applications	Components	Scoring Range
VAS (Visual Analog Scale)	Older children	tool for assessing anxiety related to hospitalization and to needle-related, diagnostic, and therapeutic procedures.	Subjective, 10 cm line labeled with descriptions of emotional states ranging from ‘I don’t feel anxious at all’ to ‘the most anxious I can imagine	0–10
FAS (Faces Anxiety Scale)	Older children	tool for assessing anxiety related to hospitalization and to needle-related, diagnostic, and therapeutic procedures.	Nine faces representing different emotional states	0–9
ICC (Induction Compliance Checklist)	Younger and older children	behavior of children during anesthesia induction	Checklist of ten items that capture negative behaviors observed during induction, such as crying, physical resistance, or attempts to escape	0–10
PAB (Pediatric Anesthesia Behavior Scale)	Younger and older children	anxiety during anesthesia induction	Behaviors as happy, sad, or frantic	3–9
HARD±	Younger and older children	assessing the severity of anxiety prior to procedures, e.g., dental treatments	10 or 15 questions, child’s emotional state across domains such as happiness, relaxation, anxiety, and worry	0–50 or 0–75
m-YPASS (Modified Yale Preoperative Anxiety Scale)	Newborns and young children	assessing procedural or preoperative anxiety	22 items divided into five behavioral domains: activity, vocalization, emotional expressivity, state of arousal, and interactions with family members	anxiety level is defined as a score of 30 or higher
OSBD (Observation Scale of Behavioral Distress)	Younger children	assess children’s behavioral responses during medical procedures	11 behavioral items, which are summed and then divided by the duration of the procedure to yield a distress score	Weights are assigned to each observed behavior
STAIC (State- Trait Anxiety Inventory-Children)	Older children	perioperative anxiety	20 items assessing state anxiety (situational, transient anxiety) and 20 items assessing trait anxiety, reflecting a general tendency to experience fear and anxiety	0–60 interpretation is based on the appropriate percentiles

## Data Availability

No new data were created or analyzed in this study. Data sharing is not applicable to this article.

## Abbreviations

COMFORT	Scale for the assessment of pain, stress and sedation levels in children
COMFORT-B	A modified, behavior- based version of the COMFORT Scale
CRIES	Crying, Requires O_2_, Increased vital sings, Expression, Sleepiness
DESS	Douleur Elefant Scan Salvadour Scale
FAS	Faces Anxiety Scale
FLACC	Face, Leg, Activity, Cry, Consolability Scale
HARD±	Heightened Anxiety Rating for Dentistry
HRV	Heart Rate Variability
ICC	Induction Compliance Checklist
m-YPAS	Modified Yale Preoperative Anxiety Scale
m-YPAS-SF	Modified Yale Preoperative Anxiety Short Form Scale
NCCPC-R	Non-Communicating Children’s Pain Check List, Revised Scale
NFCS	Neonatal Facial Coding System
NIPE	Newborn Infant Parasympathetic Evaluation monitor
N-PASS	Neonatal Pain, Agitation and Sedation Scale
NRS	Numerical Rating Scale
OSBD	Observation Scale of Behavioral Distress
PAB	Pediatric Anesthesia Behavior
PAT	Pain Assessment Tool
PIPP	Premature Infant Pain Profile
PNS	Parasympathetic Nervous System
PPP	Pediatric Pain Profile
R-FLACC	Revised FLACC
SCA	Skin Conductance Algesimeter
STAI	State-Train Anxiety Inventory
STAIC	State-Train Anxiety Inventory—Children
SpO_2_	oxygen saturation
VAS	Visual Analog Scale

## References

[1] Danel J.K., Rosada-Kurasinska J., Copik M.M., Zdanowski S., Gola W., Misiołek H., Bartkowska-Śniatkowska A., Białka S. (2025). Objective monitoring of acute pain and nociception in anaesthesia and intensive care: Evidence and applications. Anaesthesiol. Intensive Ther..

[2] Cettler M., Zielińska M., Rosada-Kurasińska J., Kubica-Cielińska A., Jarosz K., Bartkowska-Śniatkowska A. (2022). Guidelines for treatment of acute pain in children—The consensus statement of the Section of Paediatric Anaesthesiology and Intensive Therapy of the Polish Society of Anaesthesiology and Intensive Therapy. Anaesthesiol. Intensive Ther..

[3] Szafrańska A., Fonfara A., Dzudziak A., Królak-Olejnik B. (2023). Obiektywizacja oceny komfortu noworodków hospitalizowanych w oddziale intensywnej terapii—Zastosowanie NIPE. Postępy Neonatol..

[4] Bomersbach A., Sochocka L. (2018). Assessment of the intensity of procedural pain in newborns treated in the intensive care unit measured by the Neonatal Infant Pain Scale (NIPS). Prog. Health Sci..

[5] Glenzel L., do Nascimento Oliveira P., Marchi B.S., Ceccon R.F., Moran C.A. (2023). Validity and Reliability of Pain and Behavioral Scales for Preterm Infants: A Systematic Review. Pain Manag. Nurs..

[6] Zeng Z. (2022). Assessment of neonatal pain: Uni- and multidimensional evaluation scales. Front. Nurs..

[7] Świątoniowska N., Maj A., Rozensztrauch A. (2017). PAT SCORE—The tool for assessing pain intensity in newborns. J. Educ. Health Sport.

[8] Campbell-Yeo M., Eriksson M., Benoit B. (2022). Assessment and management of pain in preterm infants: A practice update. Children.

[9] Fortney A.C., Sealschott S.D., Pickler R.H. (2020). Behavioural observation of infants with life-threatening or life-limiting illness in the neonatal intensive care unit. Nurs. Res..

[10] Ista E., van Dijk M., Tibboel D., de Hoog M. (2005). Assessment of sedation levels in paediatric intensive care patients can be improved by using the COMFORT behaviour scale. Pediatr. Crit. Care Med..

[11] Michalska E., Tsupruk M. (2018). Pain management in paediatric critical care—Challenges in assessment and treatment of pain. Standardy Med..

[12] Popowicz H., Mędrzycka-Dąbrowska W., Kwiecień-Jaguś K. (2018). Prevention and treatment of pain in the neonatal intensive care unit. Ból.

[13] Bhalla T., Shepherd E., Tobias J.D. (2014). Neonatal pain management: Review. Saudi J. Anaesth..

[14] Benvenuto S., Trombetta A., Barbi E. (2022). A pragmatic approach to assessment of chronic and acute pain in children with severe neurological impairment. Children.

[15] Johansson M., Kokinsky E. (2009). The COMFORT behavioural scale and the modified FLACC scale in paediatric intensive care. Nurs. Crit. Care.

[16] El-Tallawy S.N., Ahmed R.S., Nagiub M.S. (2023). Pain management in the most vulnerable intellectual disability populations: Review. Pain Ther..

[17] Breau L.M., Burkitt C. (2009). Assessing pain in children with intellectual disabilities. Pain Res. Manag..

[18] Michalska A., Szerla M.K., Przysło Ł., Dudek J., Ortenburger D.E. (2017). Pain assessment in the course of cerebral palsy—A review of methods used in the practice of a physiotherapist, doctor and psychologist. Med. Rehabil..

[19] Nagarwal P., Rana V., Srivastava N., Kaushik N., Pruthi T.P. (2023). Reliability of three pain assessment tools in children requiring dental treatment: A comparative clinical study. J. Indian Soc. Pedod. Prev. Dent..

[20] Sansone L., Gentile C., Grasso E.A., Di Ludovico A., La Bella S., Chiarelli F., Breda L. (2023). Pain Evaluation and Treatment in Children: A Practical Approach. Children.

[21] Kimball H., Cobham V.E., Sanders M., Douglas T. (2023). Procedural anxiety among children and adolescents with cystic fibrosis and their parents. Pediatr. Pulmonol..

[22] Hettema J.M., Neale M.C., Kendler K.S. (2001). A review and meta-analysis of the genetic epidemiology of anxiety disorders. Am. J. Psychiatry.

[23] Freidl E.K., Stroeh O.M., Elkins R.M., Steinberg E., Albano A.M., Rynn M. (2017). Assessment and treatment of anxiety among children and adolescents. Focus.

[24] Yun R., Kennedy K.M., Caruso T.J. (2022). Perioperative Pediatric Anxiety: A Cry for Universal Scale Adoption. Pediatr. Qual. Saf..

[25] Kain Z.N., Wang S.M., Mayes L.C., Caramico L.A. (1999). Distress during induction of anesthesia and postoperative behavioral outcomes in children: A prospective cohort study. Anesth. Analg..

[26] Eijlers R., Staals L.M., Legerstee J.S., Berghmans J.M., Strabbing E.M., van der Schroeff M.P., Wijnen R.M.H., Kind L.S., Hillegers M.H.J., Dierckx B. (2021). Predicting Intense Levels of Child Anxiety During Anesthesia Induction at Hospital Arrival. J. Clin. Psychol. Med. Settings.

[27] Vieco-García A., López-Picado A., Fuentes M., Francisco-González L., Joyanes B., Soto C., de la Aldea A.G., Gonzalez-Perrino C., Aleo E. (2021). Comparison of different scales for the evaluation of anxiety and compliance with anesthetic induction in children undergoing scheduled major outpatient surgery. Perioper. Med..

[28] Cesare M., D’ Agostino F., Damiani G., Nurchis M.C., Ricciardi W., Cocchieri A., Nursing Public Health Group (2025). Exploring the Impact of Medical Complexity on Nursing Complexity of Care in Paediatric Patients: A Retrospective Observational Study. J. Clin. Nurs..

[29] Szamsi A. (2025). Bariers and facilitators of pain management in children: A scoping review. BMC Anesthesiol..

